# Expression and Immune Characterization of Major Histocompatibility Complex in *Paralichthys olivaceus* after Antigen Stimulation

**DOI:** 10.3390/biology12121464

**Published:** 2023-11-24

**Authors:** Jing Xing, Zhaoxia An, Xiaoqian Tang, Xiuzhen Sheng, Heng Chi, Wenbin Zhan

**Affiliations:** 1 Laboratory of Pathology and Immunology of Aquatic Animals, Key Laboratory of Mariculture, Ministry of Education (KLMME), Ocean University of China, 5 Yushan Road, Qingdao 266003, China; xingjing@ouc.edu.cn (J.X.); 17615323621@163.com (Z.A.); tangxq@ouc.edu.cn (X.T.); xzsheng@ouc.edu.cn (X.S.); chiheng@ouc.edu.cn (H.C.); 2 Laboratory for Marine Fisheries Science and Food Production Processes, Qingdao National Laboratory for Marine Science and Technology, Qingdao 266071, China

**Keywords:** flounder (*Paralichthys olivaceus*), MhcIIα, MhcIIβ, expression, immune response

## Abstract

**Simple Summary:**

The Major histocompatibility complex (Mhc) plays an essential role in antigen presentation as part of the adaptive immune system. To investigate the role of MhcII in adaptive immunity, this study cloned the *mhcIIα* and *mhcIIβ* of flounder (*Paralichthys olivaceus*) and examined their distribution and expression patterns, which varied by antigen stimulation or pathogens infection. The analyses showed that they were significantly expressed in gills, spleen, and peripheral blood leukocytes (PBLs), and MhcII molecules were co-localized with CD83 and IgM on leukocytes, respectively. The expression of both *mhcIIα* and *mhcIIβ* were significantly upregulated in flounder after infection. The percentages of MhcII^+^ cells, MhcII^+^/CD83^+^, and MhcII^+^/IgM^+^ cells increased significantly after PHA and ConA stimulation, respectively; they varied significantly in PBLs after polyI:C stimulation and no obvious variations were found after LPS treatment. These results suggested that MhcII, mainly expressed in B cells and dendritic cells, responded significantly to exogenous antigens and T cell-dependent antigens. In conclusion, MhcII was associated with cellular immunity in teleosts.

**Abstract:**

The Major histocompatibility complex (Mhc) is an important molecule for antigen presenting and binds to T cell receptors, activating T lymphocytes and triggering specific immune responses. To investigate the role of MhcII in adaptive immunity, in this study, *mhcIIα* and *mhcIIβ* of flounder (*Paralichthys olivaceus*) were cloned, polyclonal antibodies (Abs) against their extracellular regions were produced, respectively, and their distribution on cells and tissues and expression patterns, which varied by antigen stimulation or pathogen infection, were investigated. The results showed that the open reading frame (ORF) of *mhcIIα* is 708 bp, including 235 amino acids (aa); and the ORF of *mhcIIβ* is 741 bp, encoding 246aa. The *mhcIIα* and *mhcIIβ* were significantly expressed in gills, spleen, and peripheral blood leukocytes (PBLs). Their antibodies could specifically recognize eukaryotic expressed MhcIIα and MhcIIβ. MhcIIα^+^ and MhcIIβ^+^ cells were 30.2 ± 2.9% of the percentage in peripheral blood leukocytes. MhcII molecules were co-localized with CD83 and IgM on leukocytes, respectively, but not on CD4^+^ or CD8^+^ T lymphocyte subpopulations. The expression of both *mhcIIα* and *mhcIIβ* were significantly upregulated in flounder after bacteria and virus challenges. The percentages of MhcII^+^ cells, MhcII^+^/CD83^+^, and MhcII^+^/IgM^+^ double-positive cells increased significantly after PHA and ConA stimulation, respectively; they varied significantly in PBLs after polyI:C stimulation, and no variations were found after LPS treatment. In the meantime, variations in MhcII^+^ cells were consistent with that of CD4^+^ T lymphocytes. These results suggest that MhcII, mainly expressed in B cells and dendritic cells, play an essential role in antigen presentation, and respond significantly to exogenous antigens and T cell-dependent antigens. These results may provide an important reference for the study of cellular immunity in teleosts.

## 1. Introduction

The Major histocompatibility complex (Mhc) is an important molecule for antigen presenting and binds to T cell receptors, activating T lymphocytes and triggering specific immune responses. In mammals, MhcII molecules have been shown to mediate the presentation of exogenous antigens and are mainly expressed on antigen-presenting cells (APCs), while most studies on fish MhcII have focused on the genetic level [1,2].

MhcII plays a crucial role in fish adaptive immunity; it is characterized by gene cloning, bioinformatics, and expression patterns [3,4,5]. Rarely has the biological role of MhcII been discussed. This gene has been cloned and identified in Chinese longsnout catfish (*Leiocassis longirostris*), Whitespotted bambooshark (*Chiloscyllium plagiosum*), swamp eel (*Monopterus albus*), stone flounder (*Kareius bicoloratus*), and loach (*Misgurnus anguillicaudatus*) [6,7,8,9,10]. Afterward, the expressions and distributions of *mhcIIα* and *mhcIIβ* in several tissues in large yellow croaker (*Pseudosciaena crocea*), Nile tilapia (*Oreochromis niloticus*), and spotted halibut (*Verasper variegatus*), etc. were reported [11,12,13]. The findings demonstrated that the gills, spleen, and kidney had significantly high levels of *mhcIIα* and *mhcIIβ* expression. It has been shown that *mhcIIα* and *mhcIIβ* genes are significantly upregulated in the kidney of miiuy croaker (*Miichthys miiuy*) after infection of *Vibrio anguillarum* and decrease slowly after reaching a peak at 12 h [14]. After treatment with *V. anguillarum* and polyI:C, the *mhcIIα* and *mhcIIβ* genes in the head kidney and spleen of Chinese sturgeon (*Acipenser sinensis*) are highly upregulated [15]. In loach (*Misgurnus anguillicaudatus*), *mhcIIα* and *mhcIIβ* genes are significantly upregulated in the gills and spleen after infection of *Flavobacterium cloumnare* G4 and *Ichthyophthirius multifiliis* [10]. In addition, more studies on the expression of *mhcII* in Sea Perch (*Lateolabrax japonicus*), red sea bream (*Chrysophrys major*), and Atlantic salmon (*Salmo salar*) after infection were carried out [16,17,18].

MhcII molecules, which are usually polymorphic and mainly involved in antigen presentation [19,20], have been found to be distributed on certain immune cells, mainly APCs [21]. In addition, after stimulation with CpG oligonucleotide (ODN), an MhcII^+^ cell subpopulation upregulated CD86 and produced high levels of tumor necrosis factor (TNF) mRNA, suggesting that these subpopulations of MhcII^+^ cells are specialized APCs associated with macrophages and dendritic cells (DCs) [22]. MhcII molecules contain peptide-binding regions (PBRs) that are responsible for antigen recognition, and the binding of PBRs to antigenic peptides is necessary for the generation of the immune cascade, thus placing MhcII at the center of the immune response [23].

Flounder belongs to an important species of marine culture in northern China, which has high economic value, and the frequent occurrence of diseases has greatly impeded the healthy development of flounder aquaculture; our laboratory has been studying flounder for many years; it will be helpful to provide new ideas and methods for the in-depth study of MhcII molecules. In our previous research, antibodies against the dendritic cell surface antigen CD83, the T cell antigens CD4-1, CD4-2, and CD8β, and the B cell antigen IgM were generated, respectively [24,25,26,27]. In this paper, *mhcIIα* and *mhcIIβ* genes in flounder (*Paralichthys olivaceus*) were cloned, and polyclonal antibodies (Abs) against their extracellular region were produced, respectively. Their distribution on cells and tissues was analyzed, and they were defined by their co-localization on T and B cells. In addition, the expression patterns that varied via antigen stimulation or pathogens infection were investigated. The expression of MhcII and its immunological characterization in flounder after antigen stimulation may provide a reference for the cellular immunity of teleost fish.

## 2. Materials and Methods

### 2.1. Animals

An aquafarm in Rizhao, Shandong, China, supplied healthy flounder. The flounder were used for this study after two weeks of acclimation in seawater (salinity of 35‰, pH of 8.7, and depth of 1 m) that circulated (flow rate of 500 L/h) at 20 ± 2 °C. The bait used for flounder breeding was full-value compound feed, which was fed to the flounder twice a day (9:00 a.m. and 17:00 p.m.), and the amount of each feeding was 3% of the flounder’s body weight. There were 14 h of light and 10 h of darkness per day (14 L-10 D). For in vitro leukocyte production and cell stimulation tests, flounder between 40 cm and 45 cm in length were employed. Flounder in size of length 20–25 cm were used for RNA extraction and injection experiments in vivo.

The Qingdao Animal Experimental Center (Shandong, China) provided Balb/C mice and New Zealand white rabbits to produce the Abs.

The flounder were anesthetized with 60 mg/mL MS-222 for 5 min prior to sample collection. Balb/C mice and New Zealand white rabbits were anesthetized with Ethyl ether prior to sample collection. The flounder were euthanized via liquid nitrogen cryogenic freezing. The Balb/C mice were euthanized via the cervical dislocation method. The New Zealand white rabbits were euthanized via bloodletting. The protocols outlined in the Ocean University of China’s Guide for the Use of Experimental Animals were strictly followed throughout the study in compliance with the International Guiding Principles for Biomedical Research Involving Animals (EU84 2010/63). We used every tool at our disposal to lessen suffering.

### 2.2. Preparation of Leukocytes, mRNA, and HEK293 Cells

A previously published procedure was used to separate the flounders’ leukocytes from the gills, peripheral blood, head kidney, and spleen [27]. Briefly stated, the caudal vein was used to draw peripheral blood, which was then diluted with solution (65% RPMI-1640 including 20 IU mL^−1^ heparin, 0.1% *w*/*v* NaN3, and 1% *w*/*v* BSA). The gills, spleen, and kidney were removed; the gills were immersed in trypsin solution for 30 min at 37 °C, and then the gills, spleen, and kidney were squeezed via a nylon mesh filter with 65% RPMI-1640 solution to prepare cell suspensions. After centrifugation, the supernatants were spread over a discontinuous Percoll density gradient that ranged from 1.020 g/cm^3^ to 1.070 g/cm^3^. The Percoll interface’s leukocyte layers were collected after centrifugation.

The mRNA in the muscle, skin, liver, intestine, head kidney, spleen, gills, peripheral blood leukocytes (PBLs), gill leukocytes (GLs), spleen leukocytes (SPLs), and head kidney leukocytes (HKLs) were collected from nine flounder, and three flounder were randomly combined as a separate sample, respectively. The Trizol technique was used to extract the total RNA, as directed by the manufacturer. A NanoDrop-8000 spectrophotometer from Thermo Scientific (Waltham, MA, USA) was used to determine the amount and concentration of the RNA. The cDNA was created utilizing the Reverse Transcriptase M-MLV kit (TaKaRa, Dalian, China), as directed by the manufacturer. The spleen cDNA was used as a template in *mhcIIα* and *mhcIIβ* cloned genes. The cDNAs of the muscle, skin, liver, intestine, head kidney, spleen, gills, PBLs, HKLs, SPLs, and GLs were used as templates; three replicate experiments were performed for each individual sample in qPCR.

HEK-293 cells were previously frozen in the laboratory, inoculated in a 25 cm^2^ cell culture flask, and cultured in MEM medium with 20% FBS, 100 IU/mL penicillin, and 100 µg/mL streptomycin.

### 2.3. Antibodies

Specific mouse monoclonal antibodies (mAbs) against flounder CD4-1, CD4-2, and IgM, specific rabbit polyclonal Abs against flounder CD83, and specific mouse polyclonal Abs against flounder CD8β were already stored in our laboratory [24,25,26,27]. Specific rabbit and mouse polyclonal Abs against flounder MhcIIα and MhcIIβ were produced in this study. For utilization in flow cytometry and immunofluorescence staining, respectively, all Abs were diluted 1000 times in PBS.

### 2.4. Cloning of mhcIIα and mhcIIβ

The *mhcIIα* and *mhcIIβ* mRNA sequences for flounder were acquired from the NCBI database by utilizing accession numbers AY997530 and AY848955.1, respectively. The cDNA of the spleen concentration was adjusted to 5 ng/µL and used as the amplification template for the full-length of *mhcIIα* and *mhcIIβ* for gene cloning under the subsequent RT-PCR procedures: 95 °C, 5 min, (95 °C, 30 s → 58.5 °C, 30 s) × 35 cycles, 72 °C, 50 s, and 72 °C, 10 min. Amplification primers for the extracellular region of *mhcIIα* and *mhcIIβ* were designed based on the cloned full-length genes. Table 1 lists all the primers utilized in this investigation.

### 2.5. Sequence Analysis

DNAMAN was used to evaluate the full-length *mhcIIα* and *mhcIIβ* cDNA. And the protein’s biological traits were explored. Utilizing the SMART program (http://smart.embl-heidelberg.de/) (accessed on 6 January 2022), the transmembrane domain, signal peptide, and conserved protein domain were examined. The MhcIIα and MhcIIβ three-dimensional models were created by Swiss-Model (http://swissmodel.expasy.org/) (accessed on 6 January 2022). The nucleotide and protein sequences homology of flounder MhcIIα (fMhcIIα) and MhcIIβ (fMhcIIβ) were analyzed using BLAST (http://www.ncbi.nlm.nih.gov/blast) (accessed on 6 January 2022). The multiple amino acid sequence alignment was carried out by DNAMAN. Finally, the phylogenetic trees were created and examined utilizing iqtree-1.6.12 and the 1000 bootstrap attempts of the maxima likelihood method.

### 2.6. Production of Anti-Flounder MhcIIα and MhcIIβ Abs

The PCR products of *mhcIIα* and *mhcIIβ* extracellular region were purified and introduced into procaryotic expression vectors pET-32a(+). To produce recombinant MhcIIα (rMhcIIα) and MhcIIβ (rMhcIIβ), pET-32a-*mhcIIα* and pET-32a-*mhcIIβ* recombinant plasmids were transferred into *E. coli* Transetta (DE3) (Takara, Kusatsu, Japan) and stimulated with IPTG for 6 h at 30 °C during exponential growth. Following the manufacturer’s instructions, His Trap™ HP Ni-Agarose (GE Healthcare, Shanghai, China) was used to affinity-purify recombinant proteins. Purified rMhcIIα and rMhcIIβ were tested by SDS-PAGE detection, dye Coomassie brilliant blue R-250. Purified protein concentrations were calculated utilizing the Bradford process.

According to earlier procedures, the rMhcIIα and rMhcIIβ proteins were utilized to immunize mice and rabbits, respectively [26]. Their serum was concentrated and purified using protein G agarose affinity chromatography (Thermo Scientific, Waltham, MA, USA), and their polyclonal Abs against flounder MhcIIα and MhcIIβ were produced. The Abs titer and specificity were then assessed using ELISA, Western blot, and indirect immunofluorescence assay (IIFA), respectively.

### 2.7. Expression of mhcIIα and mhcIIβ in Flounder

The expression levels of f*mhcIIα* and f*mhcIIβ* were measured via the LightCycler^®^ 480II Real-Time System (Roche, Basel, Switzerland) using the 2× Universal SYBR Green Fast qPCR Mix (ABclonal, Wuhan, China). The *mhcIIα* and *mhcIIβ* gene fragments were amplified utilizing specific primers (Table 1). The internal control utilized “*β-actin*”; each test was carried out in triplicate. The qPCR program was 95 °C, 3 min, (95 °C, 5 s → 60 °C, 30 s) × 40 cycles, 95 °C, 5 s, and 65 °C, 60 s. The 2^−ΔΔCt^ method was used to assess all data.

In our lab, we reserved strains of *Vibrio anguillarum*, *Edwardsiella tarda*, and *Hirame rhabdovirus* (HIRRV). In the infection experiment, bacterial (1.0 × 10^8^ CFU/mL) and HIRRV suspensions (1.0 × 10^5^ TCID_50_) were employed. A total of 252 flounder were arbitrarily split into four groups, and the flounder were injected with 100 µL of PBS, *V. anguillarum*, *E. tarda*, and HIRRV in the abdominal cavity, respectively. Nine fish were included in each group; the PBLs, HKLs, SPLs, and GLs were randomly selected at 12, 24, 36, 48, 72, and 96 h, and every three fish randomly selected were combined as a separate sample and then used for RNA extraction. qPCR was utilized to ascertain the modulation of f*mhcIIα* and f*mhcIIβ* expression after infection. Three replicate experiments were performed for each individual sample.

### 2.8. Eukaryotic Transfection

The full-length *mhcIIα* and *mhcIIβ* PCR products were purified and cloned into pTag-EGFP eukaryotic expression vectors, respectively. Recombinant pTag-EGFP-*mhcIIα* and pTag-EGFP-*mhcIIβ* plasmids were sequenced and then transformed into *E. coli* Trelief™ 5α, respectively. The plasmids pTag-EGFP, pTag-EGFP-*mhcIIα,* and pTag-EGFP-*mhcIIβ* were each transfected into HEK-293 cells that had been grown in minimum essential medium (DMEM) using Lipo8000™ Transfection Reagent (Beyotime, Shanghai, China), and after 48 h, a significant amount of green fluorescence was visible, indicating successful transfection. The transfected HEK-293 cells were collected and used for Western blot and IIFA.

### 2.9. Western Blot

Lysates from transfected HEK-293 cells with pTag-EGFP-*mhcIIα* and pTag-EGFP-*mhcIIβ* and the purified rMhcIIα and rMhcIIβ were spread on SDS-PAGE and transferred to PVDF membranes (Merck Millipore, Taufkirchen, Germany). The membranes were then closed with PBS, including 4% BSA, for 1 h. The membranes containing rMhcIIα and rMhcIIβ proteins were treated with MhcIIα and MhcIIβ Abs to be tested for 1.5 h, respectively, to identify the crossover of MhcIIα and MhcIIβ Abs. The membranes, including pTag-EGFP-*mhcIIα* and pTag-EGFP-*mhcIIβ,* transfected HEK-293 cells lysates were incubated with MhcIIα and MhcIIβ Abs for 1.5 h, respectively, to identify the specificity of MhcIIα and MhcIIβ Abs. Serums from no immunized rabbits and mice were used as controls, respectively. Then, PBST (PBS with 0.5% Tween 20) was used to wash three times. Antibodies bindings were tested using HRP Goat–anti-rabbit and Goat–anti-mouse IgG-alkaline phosphatase conjugate (dilution with PBS 1:5000) (Sigma, St. Louis, MO, USA) for 45 min and three PBST washes. The membranes were then dyed using the most recent HRP dye (Thermo Scientific, Waltham, MA, USA) (All incubations were carried out at 37 °C).

### 2.10. Immunofluorescence Staining (IF)

The transfected HEK-293 cells (5 × 10^6^ cells/mL) and PBLs (5 × 10^6^ cells/mL) were settled onto slides at room temperature for 2 h and were blocked with 5% BSA for 1 h after being fixed for 15 min with 4% paraformaldehyde fixative. The transfected HEK-293 cells were treated in the dark for 1.5 h with MhcIIα and MhcIIβ Abs, respectively. The negative control was composed of serum from unvaccinated rabbits and mice that took the place of Abs as the primary antibody. Then, in three PBST washes, cells were incubated with Alexa Fluor Cy3-conjugated goat anti-rabbit and anti-mouse IgG, respectively, for 45 min in the dark. The compound of rabbit–anti-flounder MhcIIα and MhcIIβ Abs (MhcII rabbit Abs) were mixed with CD4-1, CD4-2, and IgM mAbs, as well as CD8β Abs, respectively. The compound of mouse–anti-flounder MhcIIα and MhcIIβ Abs (MhcII mouse Abs) were mixed with CD83 Abs. Those mixed Abs were incubated with PBLs as primary Abs for 1.5 h; after three PBST washes, leukocytes were incubated for 45 min avoided light with a combination of FITC-conjugated goat–anti-rabbit and Alexa Fluor 649-conjugated goat–anti-mouse IgG, and with a combination of FITC-conjugated goat–anti-mouse and Alexa Fluor 649-conjugated goat–anti-rabbit IgG (All Abs from Sigma, dilution with PBS 1:1000), respectively. After three PBST washes, the transfected HEK-293 cells and PBLs were treated with DAPI (Bio-Legend, San Diego, CA, USA) for 15 min and avoided light. After the last washing, the cell-containing slides were examined under a fluorescence microscope (Olympus DP70, Tokyo, Japan) (All incubations were carried out at 37 °C).

### 2.11. Stimulation of Leukocytes by Antigens

Following the procedure outlined in our prior study, PBLs and GLs were aseptically extracted from flounder using the inconsecutive Percoll (Pharmacia, Sofia, Bulgaria) slope (1.020/1.070) [28]. Then, 1 mL of PBLs and GLs were put into each well of a 24-well culture plate to achieve a cell density of 5 × 10^6^ cells/well. Penicillin/streptomycin and 10% fetal calf serum were added to an L-15 medium for culturing the cells and then supplemented with LPS (10 µg/well), polyI:C (10 µg/well), PHA (5 µg/well), and ConA (5 µg/well). As a negative control, PBS was mixed with the cells. After stimulation for 6, 12, 24, 36, 48, and 72 h at 22 °C, the leukocytes were collected. And then, the PBLs and GLs were used for flow cytometry analysis.

### 2.12. Flow Cytometry (FCM)

To identify the expression of MhcIIα and MhcIIβ at the cellular level, MhcIIα rabbit Abs were mixed with CD4-1, CD4-2, CD8β, and IgM Abs, respectively; in addition, MhcIIβ rabbit Abs were mixed with CD4-1, CD4-2, CD8β, and IgM Abs, respectively. For detecting the expression of MhcII after stimulation in vitro, MhcII rabbit Abs were combined with IgM Abs. Mouse anti-MhcII Abs were mixed with CD83 Abs. The combination of Abs instead of primary Abs. Serum from unvaccinated rabbits or mice was utilized as a negative control. And then, cells were treated with Abs for 1.5 h. Three PBST washes later, leukocytes were exposed to the compound of FITC Goat–anti-rabbit and Alexa fluor 649 Goat–anti-mouse IgG for 45 min, avoided light, and washed again. An Accuri C6 flow cytometer (BD Accuri, Ann Arbor, MI, USA) was utilized to examine the cell suspensions.

### 2.13. Statistical Analysis

Data in this paper were provided as mean ± SD after the trials were conducted three times in duplicate. The significance level was set at *p* < 0.05. Utilizing SPSS 26.0 software, the statistical analysis included a one-way analysis of variance (ANOVA) and Duncan’s multiple comparisons.

## 3. Results

### 3.1. Characterizations of fMhcIIα and fMhcIIβ

**Figure 1:**
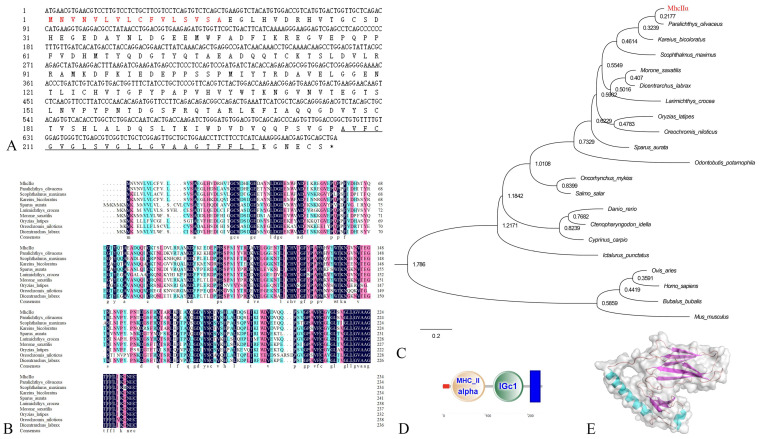
Sequence analysis of Cloned MhcIIα in flounder. (**A**): The cDNA sequence of *mhcIIα* in flounder. Red indicates the signal peptide; stop codon is shown by the *; and underlined indicates transmembrane domain. (**B**): Multiple alignments of MhcIIα in teleosts. The residues = 100% identical among the aligned sequences are in black, ≥75% identical are in red, ≥50% identical are in blue. The letters indicate amino acid sequences. The dots in the amino acid sequences indicate gaps introduced to maximize alignment. Each sequence’s accession number was as follows: *Paralichthys olivaceus* (AAY18782.1), *Scophthalmus maximus* (AAZ06134.1), *Kareius bicoloratus* (AFY98544.1), *Sparus aurata* (AAY42849.1), *Larimichthys crocea* (ABV48907.1), *Morone saxatilis* (AAA49381.1), *Oryzias latipes* (AGA53806.1), *Oreochromis niloticus* (AEO44577.1), and *Dicentrarchus labrax* (ABH09449.1). (**C**): Phylogenetic tree analysis of MhcIIα between flounder and other species. Red color indicates the MhcIIα of flounder cloned in this experiment. The accession number for each sequence was as follows: *Paralichthys olivaceus* (AAY18782.1), *Scophthalmus maximus* (AAZ06134.1), *Oncorhynchus mykiss* (CAB96452.1), *Kareius bicoloratus* (AFY98544.1), *Ictalurus punctatus* (AAD39868.1), *Sparus aurata* (AAY42849.1), *Danio rerio* (AAA16369.1), *Salmo salar* (AGH92604.1), *Cyprinus carpio* (CAA64708.1), *Larimichthys crocea* (ABV48907.1), *Morone saxatilis* (AAA49381.1), *Oryzias latipes* (AGA53806.1), *Oreochromis niloticus* (AEO44577.1), *Dicentrarchus labrax* (ABH09449.1), *Odontobutis potamophila* (ALB35651.1), *Ctenopharyngodon idella* (ABW37740.1), *Ovis aries* (ABV90473.1), *Bubalus bubalis* (ABA28748.1), *Mus musculus* (AAB46387.1), and *Homo sapiens* (QUA12595.1). (**D**): Conservative structural domain analysis of MhcIIα; red bar represents signal peptide; and blue bar represents transmembrane domain. (**E**): Tertiary structure prediction result of MhcIIα. Blue indicates α-helix, red indicates β-sheet.

**Figure 2:**
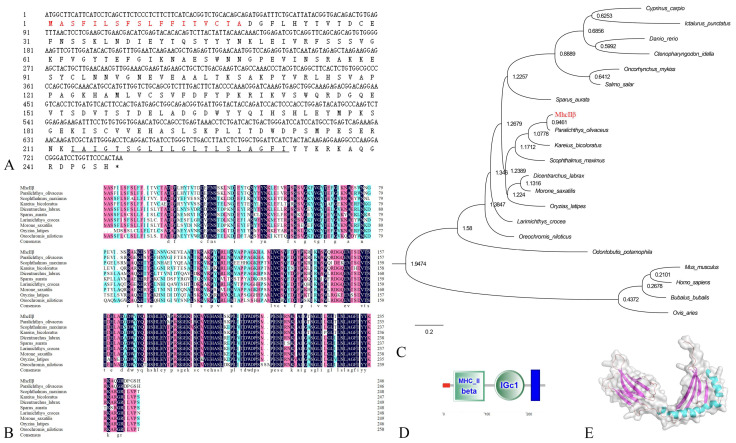
Sequence analysis of Cloned MhcIIβ in flounder. (**A**): The cDNA sequence of *mhcIIβ* in flounder. Red indicates the signal peptide; stop codon is shown by the *; and underlined indicates transmembrane domain. (**B**): Multiple alignments of MhcIIβ in teleosts. The residues = 100% identical among the aligned sequences are in black, ≥75% identical are in red, ≥50% identical are in blue. The letters indicate amino acid sequences. The dots in the amino acid sequences indicate gaps introduced to maximize alignment. Each sequence’s accession number was as follows: *Paralichthys olivaceus* (AAX52912.1), *Scophthalmus maximus* (AWO97408.1), *Kareius bicoloratus* (ADG45932.1), *Dicentrarchus labrax* (ABH09450.1), *Sparus aurata* (XP_030269046.1), *Larimichthys crocea* (ABV48909.1), *Morone saxatilis* (AAA49380.1), *Oryzias latipes* (AGA53815.1), and *Oreochromis niloticus* (AEE73592.1). (**C**): Phylogenetic tree analysis of MhcIIβ between flounder and other species. Red color indicates the MhcIIβ of flounder cloned in this experiment. The accession number for each sequence was as follows: *Paralichthys olivaceus* (AAX52912.1), *Scophthalmus maximus* (AWO97408.1), *Oncorhynchus mykiss* (AAD53026.1), *Salmo salar* (CAA49726.1), *Kareius bicoloratus* (ADG45932.1), *Cyprinus carpio* (XP_042605849.1), *Danio rerio* (NP_001007207.2), *Dicentrarchus labrax* (ABH09450.1), *Sparus aurata* (XP_030269046.1), *Ictalurus punctatus* (AAB67871.1), *Larimichthys crocea* (ABV48909.1), *Morone saxatilis* (AAA49380.1), *Oryzias latipes* (AGA53815.1), *Oreochromis niloticus* (AEE73592.1), *Odontobutis potamophila* (ALB35650.1), *Ctenopharyngodon idella* (AEM75094.1), *Ovis aries* (ABV90472.1), *Bubalus bubalis* (BAW81733.1), *Mus musculus* (AKO62645.1), and *Homo sapiens* (ACX50638.1). (**D**): Conservative structural domain analysis of MhcIIβ; red bar represents signal peptide; and blue bar represents transmembrane domain. (**E**): Tertiary structure prediction result of MhcIIβ. Blue indicates α-helix, red indicates β-sheet.

Using online biological analysis software, the fundamental sequences and structures of cloned fMhcIIα and fMhcIIβ proteins were examined. The open reading frame (ORF) length of the *mhcIIα* gene was 708 bp, encoding 235 amino acids (aa) (Figure 1A); the ORF length of the *mhcIIβ* gene was 741 bp, encoding 246 aa (Figure 2A). The fMhcIIα and fMhcIIβ have molecular weights of 25.8 kDa and 27.6 kDa and isoelectric points of 4.62 and 6.17, respectively.

According to the results of the alignment of numerous amino acid sequences, the homology of fMhcIIα and fMhcIIβ and other teleosts scopes from 60.3% to 80.9% and 64.2% to 78.0%, and the highest homology is to *Kareius bicoloratus* (80.9% and 78.0%) (Figure 1B and Figure 2B), respectively. The fMhcIIα and fMhcIIβ clusters are most closely related to *K. bicoloratus* and *Scophthalmus maximus* on the part of their genetic evolutionary position (Figure 1C and Figure 2C).

Analysis of the conserved domains shows that both fMhcIIα and fMhcIIβ are transmembrane proteins. The fMhcIIα and fMhcIIβ contain transmembrane domain (aa207-aa229 and aa213-aa232), signal peptide (aa1-aa16 and aa1-aa18), and extracellular region (aa17-aa206 and aa19-aa212), respectively. They both have an IgC domain and an MhcIIα and MhcIIβ domain (Figure 1D and Figure 2D), respectively. Then, three-dimensional fMhcIIα and fMhcIIβ models were constructed; they have a classical organization in two distinct domains, respectively. (Figure 1E and Figure 2E).

### 3.2. Tissue Distribution of mhcIIα and mhcIIβ

**Figure 3:**
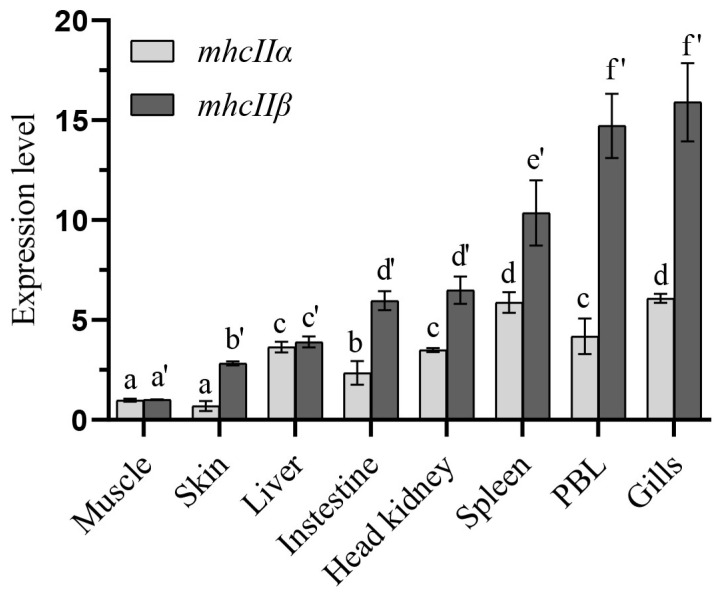
The expression level of *mhcIIα* and *mhcIIβ* genes in flounder. “*β-actin*” was used for internal control. The results are provided as means ± SD. The groups with the same letter are not significantly different from one another. (*n* = 9, *p* < 0.05).

The distribution characteristics of f*mhcIIα* and f*mhcIIβ* were detected by RT-qPCR and showed that *mhcIIα* and *mhcIIβ* mRNA were expressed in gills, PBLs, spleen, head kidney, intestine, liver, skin, and muscle in healthy flounder (Figure 3). Moreover, f*mhcIIα* and f*mhcIIβ* showed similar distribution characteristics in which significant signals were detected in the gill, spleen, PBLs, and head kidney, and poor expression was found in muscle and skin.

### 3.3. Abs Specificity of MhcIIα and MhcIIβ

**Figure 4:**
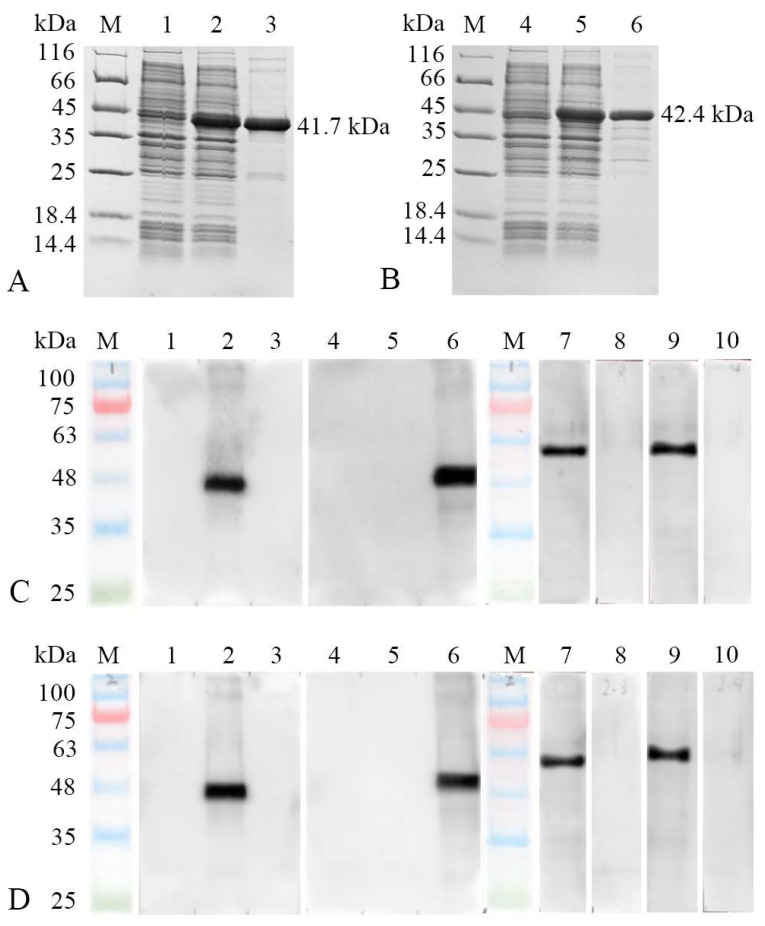
SDS-PAGE results of recombinant MhcIIα (rMhcIIα) and MhcIIβ (rMhcIIβ) and Western blot results of MhcIIα and MhcIIβ antibodies (Abs). (**A**): SDS-PAGE of rMhcIIα. Lane (L) M: Marker; L 1: transformed *E. coli* without IPTG induction; L 2: transformed *E. coli* induced with IPTG; L 3: purified rMhcIIα. (**B**): SDS-PAGE of rMhcIIβ. L M: Marker; L 4: transformed *E. coli* without IPTG induction; L 5: transformed *E. coli* induced with IPTG; L 6: purified rMhcIIβ. (**C**): Western-blot results of rabbit anti-flounder MhcIIα and MhcIIβ Abs. L M: Marker; L 1: MhcIIα Abs to pET-32a empty plasmid protein; L 2: MhcIIα Abs to rMhcIIα; L 3: MhcIIα Abs to rMhcIIβ; L 4: MhcIIβ Abs to pET-32a empty plasmid protein; L 5: MhcIIβ Abs to rMhcIIα; L 6: MhcIIβ Abs to rMhcIIβ; L 7: MhcIIα Abs to HEK293 cell lysates transfected with pTag-EGFP-*mhcIIα* recombinant plasmid; L 8: Unimmunized rabbits serum to HEK293 cell lysates transfected with pTag-EGFP-*mhcIIα* recombinant plasmid; L 9: MhcIIβ Abs to HEK293 cell lysates transfected with pTag-EGFP-*mhcIIβ* recombinant plasmid; L 10: Unimmunized rabbits serum to HEK293 cell lysates transfected with pTag-EGFP-*mhcIIβ* recombinant plasmid. (**D**): Western blot results of mouse anti-flounder MhcIIα and MhcIIβ Abs (Replaced all the rabbit anti-flounder Abs in C with the corresponding mouse anti-flounder Abs).

The SDS-PAGE revealed the pET-32a-MhcIIα and pET-32a-MhcIIβ recombinant proteins were successfully expressed and had molecular weights of 41.7 kDa and 42.4 kDa (Figure 4A,B, lane 2), respectively, which agreed with the theoretical molecular mass. In addition, high-purity rMhcIIα and rMhcIIβ proteins were detected (Figure 4A,B, lane 3), respectively. To explore the expression of MhcIIα and MhcIIβ in different cell types, specific rabbit and mouse Abs were produced. The titers of all Abs were 1:100,000 detected using ELISA.

After being transfected into the HEK293 cell line, the recombinant plasmids pTag-EGFP-MhcIIα and pTag-EGFP-MhcIIβ were examined under the fluorescent microscope after 48 h. The plasmids were successfully transfected since there was clear green fluorescence in the transfected group but negative in the untransfected group.

The MhcIIα and MhcIIβ Abs could specifically recognize about 54.8 kDa (Figure 4C,D, lane 7) and 56.6 kDa (Figure 4C,D, lane 9) bands, respectively, in HEK293 cell line lysates transfected with pTag-EGFP-*mhcIIα* and pTag-EGFP-*mhcIIβ* eukaryotic plasmid, respectively. They matched the molecular mass that was predicted. Additionally, there was no cross-reactivity among MhcIIα and MhcIIβ Abs; MhcIIα Abs could uniquely detect rMhcIIα but had no reaction to rMhcIIβ (Figure 4C,D, lane 2, 3). MhcIIβ Abs could uniquely detect rMhcIIβ but no rMhcIIα (Figure 4C,D, lane 5, 6). The negative control yielded no positive results.

**Figure 5:**
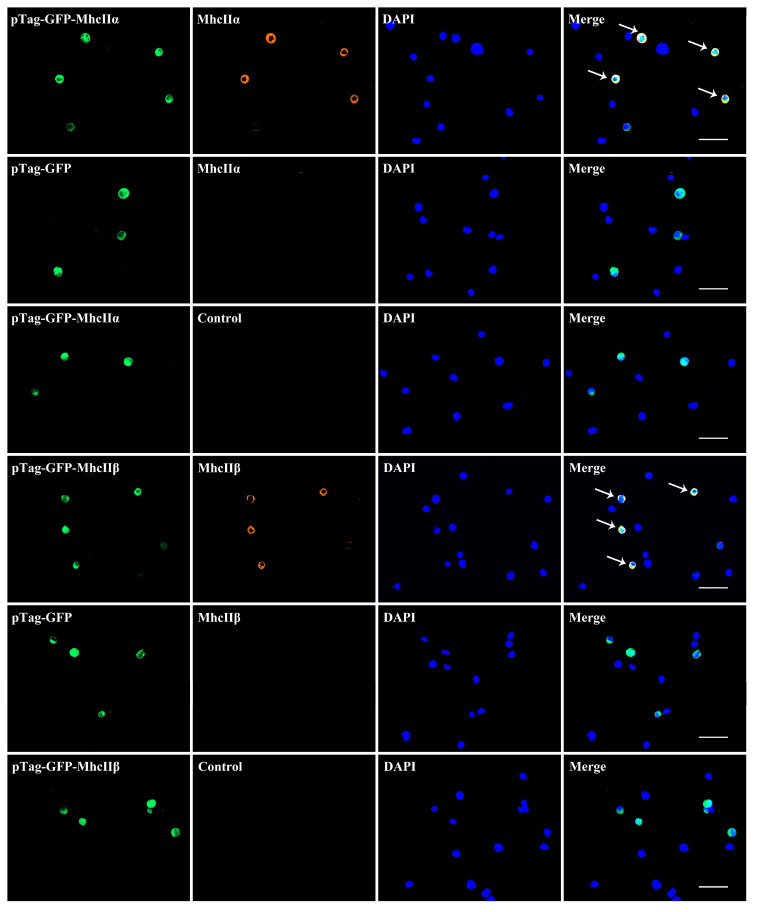
Indirect immunofluorescence results of mouse anti-flounder MhcIIα and MhcIIβ Abs and HEK293 cell lines transfected with eukaryotic plasmid. Arrows indicate double positive cells. Bar = 50 μm.

IIFA results showed that MhcIIα and MhcIIβ Abs were able to bind cells that had been transfected with pTag-EGFP-*mhcIIα* and pTag-EGFP-*mhcIIβ* eukaryotic plasmids, respectively, but not cells that had been transfected with the pTag-EGFP null plasmid. Both types of cells could not be bound by the negative serum (Figure 5 and Figure S1). According to the findings, the produced Abs have a high degree of specificity and can be applied in further experiments.

### 3.4. The Localization of MhcIIα and MhcIIβ in T and B Lymphocyte

**Figure 6:**
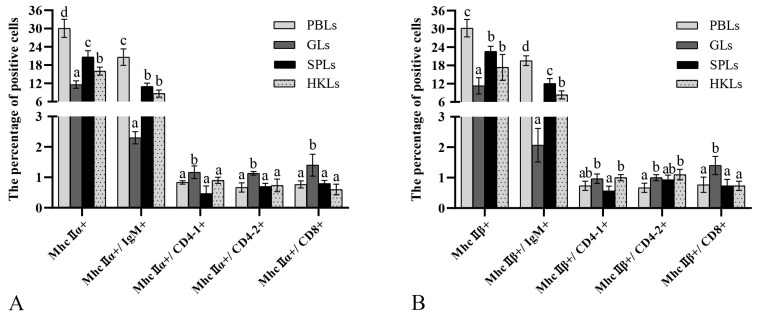
Flow cytometry results of the proportion of MhcIIα (**A**) and MhcIIβ (**B**) in T and B lymphocytes. (**A**): The percentages of MhcIIα^+^, MhcIIα^+^/IgM^+^, MhcIIα^+^/CD4-1^+^, MhcIIα^+^/CD4-2^+^ and MhcIIα^+^/CD8^+^ cells in peripheral blood, gills, head kidney, and spleen. (**B**): The percentages of MhcIIβ^+^, MhcIIβ^+^/IgM^+^, MhcIIβ^+^/CD4-1^+^, MhcIIβ^+^/CD4-2^+^ and MhcIIβ^+^/CD8^+^ cells in peripheral blood, gills, head kidney, and spleen. In control groups, unimmunized rabbit and mouse serum were used as primary antibodies. The results are provided as means ± SD. Different letters above the bar represent the statistical significance (*n* = 9, *p* < 0.05).

FCM and IIFA results showed MhcIIα^+^, MhcIIα^+^/CD4-1^+^, MhcIIα^+^/CD4-2^+^, MhcIIα^+^/IgM^+^, MhcIIα^+^/CD8^+^, MhcIIβ^+^, MhcIIβ^+^/CD4-1^+^, MhcIIβ^+^/CD4-2^+^, MhcIIβ^+^/IgM^+^, and MhcIIβ^+^/CD8^+^ cells present in peripheral blood, gills, kidney, and spleen (Figures S2 and S3). MhcIIα^+^ and MhcIIβ^+^ leukocytes with the highest percentages in peripheral blood to 30.1 ± 3.0% and 30.2 ± 2.9%, respectively. MhcIIα^+^ and MhcIIβ^+^ leukocytes did not colocalize with T lymphocyte subpopulations. Their percentages of MhcIIα^+^, MhcIIβ^+^, MhcIIα^+^/IgM^+^, and MhcIIβ^+^/IgM^+^ cells were 30.1 ± 3.0%, 30.2 ± 2.9%, 20.6 ± 2.7%, and 19.6 ± 1.6% in PBLs, 11.6 ± 1.2%, 11.3 ± 2.7%, 2.3 ± 0.2%, and 2.1 ± 0.6% in the gills, 16.0 ± 1.3%, 17.4 ± 4.2%, 8.7 ± 1.2%, and 8.4 ± 1.3% in the head kidney, 20.7 ± 2.1%, 22.6 ± 1.7%, 11.0 ± 1.1%, and 12.1 ± 1.6% in the spleen, respectively (Figure 6A,B). PBLs had higher percentages of MhcIIα^+^ and MhcIIβ^+^ leukocytes than spleen, head kidney, and gills. In addition, there were no significant variations in the percentages of both MhcIIα^+^ and MhcIIβ^+^ cells in each tissue, so the subsequent experiments were performed by mixing MhcIIα Abs and MhcIIβ Abs in a 1:1 ratio.

**Figure 7:**
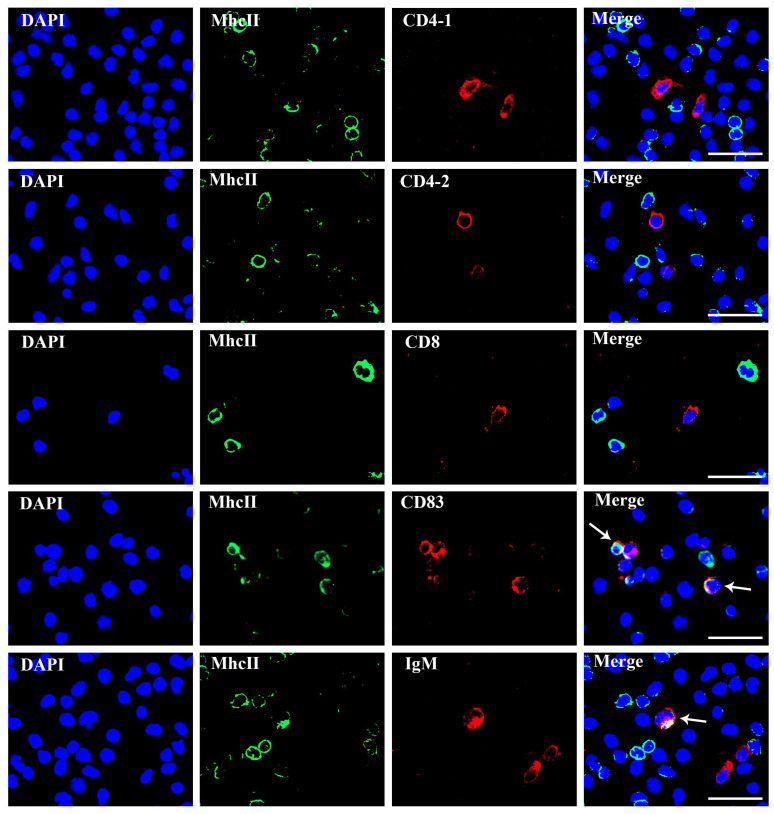
Double immunofluorescence staining results of MhcII^+^/CD4-1^+^, MhcII^+^/CD4-2^+^, MhcII^+^/CD8^+^, MhcII^+^/CD83^+^, and MhcII^+^/IgM^+^ lymphocytes. Arrows indicate double positive cells. The unimmunized serum was utilized as negative control. Bar = 20 µm.

The results of IIFA illustrated the same results as that of FCM; MhcII were co-localized with CD83 and IgM on leukocytes, respectively, but not on CD4 and CD8 T lymphocyte subpopulations (Figure 7). These findings suggested that MhcII mostly distributes on the surface of APCs.

### 3.5. Gene Expression of mhcIIα and mhcIIβ after Infection

**Figure 8:**
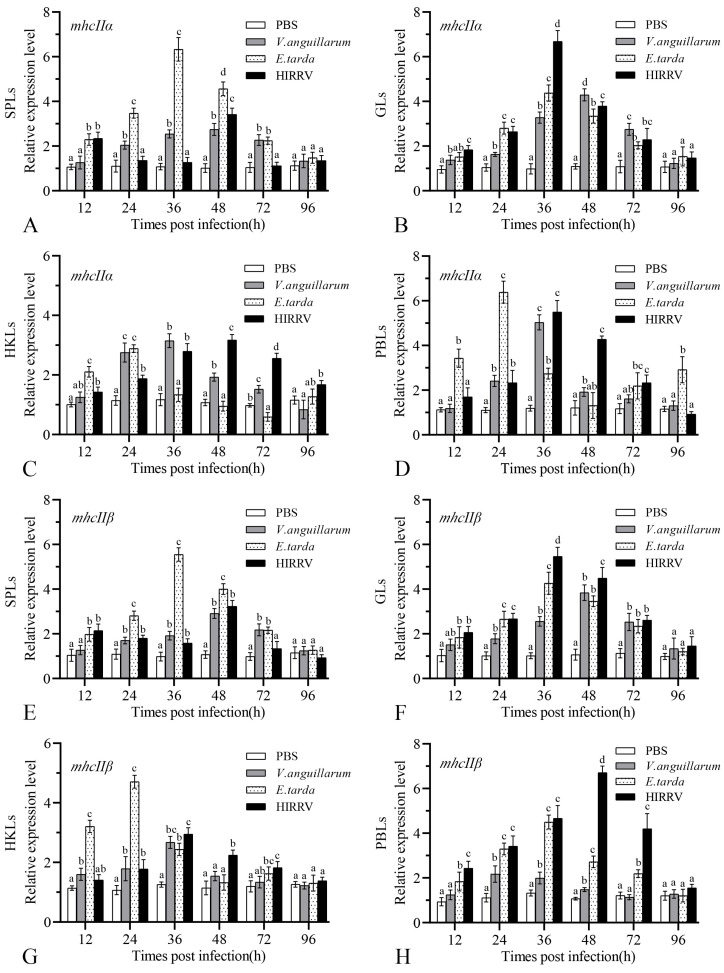
The expression of *mhcIIα* and *mhcIIβ* in spleen leukocytes (SPLs), gill leukocytes (GLs), head kidney leukocytes (HKLs), and peripheral blood leukocytes (PBLs) of flounder after injection with *Vibrio anguillarum*, *Edwardsiella tarda,* and HIRRV. The control group was injected with PBS. (**A**): *mhcIIα* in SPLs. (**B**): *mhcIIα* in GLs. (**C**): *mhcIIα* in HKLs. (**D**): *mhcIIα* in PBLs. (**E**): *mhcIIβ* in SPLs. (**F**): *mhcIIβ* in GLs. (**G**): *mhcIIβ* in HKLs. (**H**): *mhcIIβ* in PBLs. The results are provided as means ± SD. Different letters above the bar represent the statistical significance (*n* = 9, *p* < 0.05).

To determine the role of MhcIIα and MhcIIβ in bacteria and virus challenges, their expression was evaluated via qPCR in SPLs, GLs, HKLs, and PBLs at various time intervals after immunization. The findings revealed that *mhcIIα* (Figure 8A–D) and *mhcIIβ* (Figure 8E–H) genes were significantly upregulated in all tissue leukocytes of the immunized group against the PBS control group (ANOVA, *p* < 0.05), and all peaked at 24–48 h post-infection with all types of pathogens then decreased (Figure 8). In addition, *mhcIIα* and *mhcIIβ* genes showed similar trends of variation. Early *mhcIIα* and *mhcIIβ* expressions were found after *E. tarda* infection and peaked at 24 and 36 h in all tissue leukocytes, having the most significant expression levels in SPLs (Figure 8A,E). After *V. anguillarum* and HIRRV infection, the maximum expression level of *mhcIIα* and *mhcIIβ* were at 36 and 48 h, with significant expression levels in PBLs and GLs (Figure 8B,D,F,H). After *V. anguillarum* infection, the expressions of the *mhcIIα* gene in PBLs and GLs were 5.03-fold and 4.29-fold higher than that in the control group, respectively (Figure 8B,D).

### 3.6. Variations in MhcII^+^, MhcII^+^/IgM^+^, MhcII^+^/CD83^+^, and CD4^+^ Leukocytes after Antigen Stimulation

**Figure 9:**
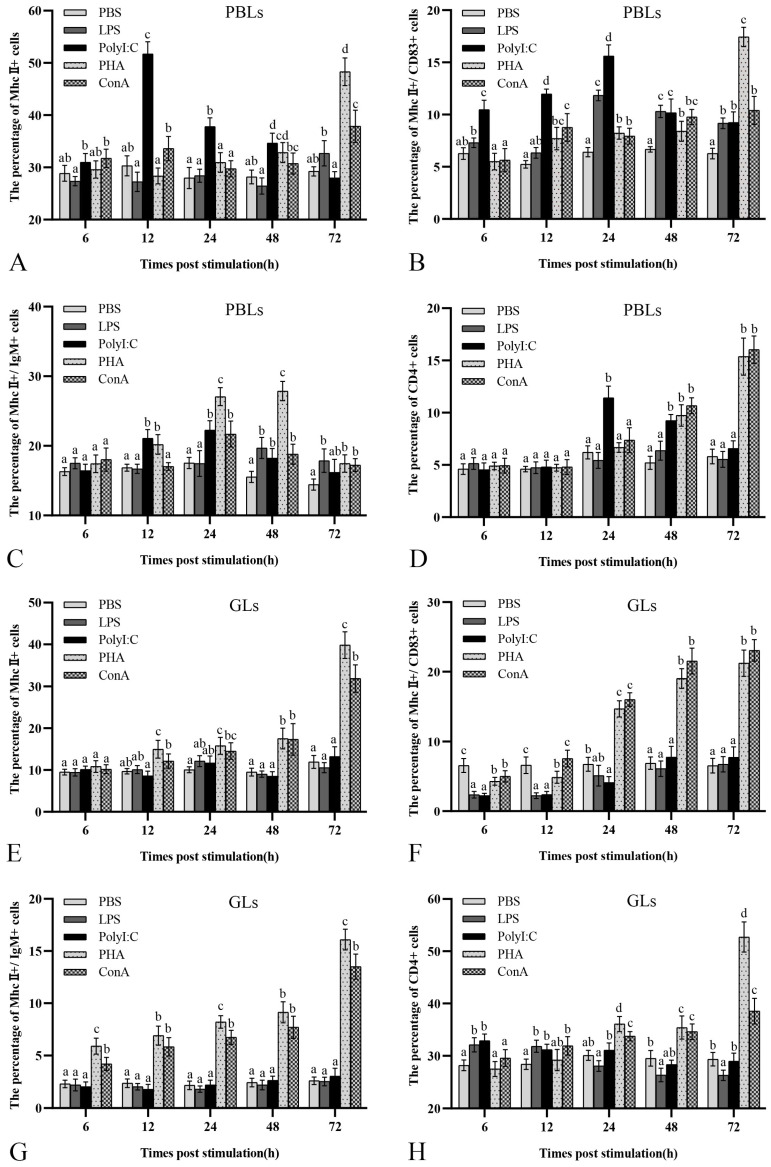
Flow cytometry analysis of the proliferation of MhcII^+^, MhcII^+^/CD83^+^, MhcII^+^/IgM^+^, and CD4^+^ cells at 6, 12, 24, 48, and 72 h after LPS, polyI:C, PHA, and ConA stimulation of flounder PBLs and GLs in vitro. PBS stimulation was used as a control group. (**A**): MhcII^+^ cells in PBLs. (**B**): MhcII^+^/CD83^+^ cells in PBLs. (**C**): MhcII^+^/IgM^+^ cells in PBLs. (**D**): CD4^+^ cells in PBLs. (**E**): MhcII^+^ cells in GLs. (**F**): MhcII^+^/CD83^+^ cells in GLs. (**G**): MhcII^+^/IgM^+^ cells in GLs. (**H**): CD4^+^ cells in GLs. The control group of unimmunized rabbit and mouse serum as primary antibody. The results are provided as means ± SD. Different letters above the bar represent the statistical significance (*n* = 9, *p* < 0.05).

After stimulation, PBLs and GLs in vitro with LPS, poly I:C, PHA, and ConA, respectively, MhcII^+^, MhcII^+^/IgM^+^, MhcII^+^/CD83^+^, and CD4^+^ cells were detected using FCM. In PBLs, the percentages of MhcII^+^, MhcII^+^/CD83^+^, MhcII^+^/IgM^+^, and CD4^+^ in the PBS group were 29.2 ± 0.9%, 16.3 ± 0.6%, 6.3 ± 0.5%, and 5.2 ± 0.6%, respectively. In the LPS group, no remarkable rise in MhcII^+^ and CD4^+^ cells (Figure 9A,D) was found in peripheral blood, but the MhcII^+^/CD83^+^ and MhcII^+^/IgM^+^ (Figure 9B,C) showed an increase, and peaked at 48 and 24 h, with proportions of 19.7 ± 1.5% and 11.8 ± 0.5%, respectively. After polyI:C stimulation, the proportions of MhcII^+^, MhcII^+^/CD83^+^, MhcII^+^/IgM^+^, and CD4^+^ cells showed increase followed by decrease, and peaked at 12, 24, 24, and 24 h, with the percentages of 51.7 ± 2.3%, 22.3 ± 1.4%, 15.6 ± 1.1%, and 11.4 ± 1.1%, respectively. The percentages of MhcII^+^, MhcII^+^/CD83^+^, and CD4^+^ cells in the PHA group exhibited a propensity to steadily rise until the end and peaked at 72 h, with the proportions of 48.3 ± 2.7%, 17.4 ± 0.7%, and 17.4 ± 0.7%, respectively. The ratios of MhcII^+^/IgM^+^ cells revealed an increase followed by a decrease and peaked at 48 h with a proportion of 27.9 ± 1.4%. The percentages of MhcII^+^ and MhcII^+^/CD83^+^ cells in the ConA group showed a similar trend, increased, and peaked at 72 h with proportions of 37.9 ± 3.1% and 10.4 ± 1.3%, respectively. The MhcII^+^/IgM^+^ cells in the ConA group showed an increase and peaked at 24 h with a percentage of 21.7 ± 1.9%, and there was a tendency for the percentage of CD4^+^ cells to gradually rise and peaked at 72 h with a proportion of 16.0 ± 1.3%. The proliferation of cells in the polyI:C, PHA, and ConA groups was more significant than that in the LPS group, and the cellular response to polyI:C stimulation was much more rapid (Figure 9A–D).

In GLs, the proportions of MhcII^+^, MhcII^+^/IgM^+^, MhcII^+^/CD83^+^, and CD4^+^ cells in the PBS group were 11.9 ± 1.5%, 2.6 ± 0.4%, 6.5 ± 1.1%, and 29.4 ± 1.3%, respectively; there were no significant proliferative responses of MhcII^+^ and MhcII^+^/IgM^+^ cells (Figure 9E,G) after LPS and polyI:C stimulation; the proportion of MhcII^+^/CD83^+^ cells (Figure 9F) showed a rapid decrease but the CD4^+^ cells (Figure 9H) showed a significant increase at 6–12 h. After PHA and ConA stimulation, MhcII^+^, MhcII^+^/IgM^+^, MhcII^+^/CD83^+^, and CD4^+^ cells proliferated significantly (ANOVA, *p* < 0.05), exhibited a propensity to rise progressively until the end of the sample period, and all of them peaked at 72 h; in the PHA group, the percentages of MhcII^+^, MhcII^+^/IgM^+^, MhcII^+^/CD83^+^, and CD4^+^ cells were 39.8 ± 3.2%, 16.1 ± 1.0%, 21.2 ± 1.9%, and 52.7 ± 2.9%, respectively; and in the ConA group the proportions of MhcII^+^, MhcII^+^/IgM^+^, MhcII^+^/CD83^+^, and CD4^+^ cells were 31.9 ± 3.2%, 13.5 ± 1.2%, 23.1 ± 1.6%, and 38.5 ± 2.4%, respectively (Figure 9E–H).

## 4. Discussion

Sequences from fMhcIIα and fMhcIIβ were clone and examined for structural domains in this experiment. The protein’s three-dimensional models showed a classical structure in two distinct domains, which resemble the Chinese longsnout catfish [6]. The MhcIIα and MhcIIβ molecules are transmembrane proteins with an immunoglobulin-like structural domain similar to human MhcIIα and MhcIIβ [29]. Utilizing the maxima likelihood method, phylogenetic research revealed that fMhcIIα and fMhcIIβ were clustered with stone flounder (*Kareius bicoloratus*) and turbot (*S. maximus*) (Figure 1C and Figure 2C). The homology with stone flounder were 80.9% and 78.0% (Figure 1B and Figure 2B), respectively, which were the highest. They are distantly related to mammalian MhcIIα and MhcIIβ. This may be because flounder and stone flounder share a common taxonomic status and have undergone conservative gene evolution [9,30].

Tissue distribution analysis showed that *mhcIIα* and *mhcIIβ* were primarily discovered in gills, spleen, and PBLs; these were immune-associated tissues (Figure 3). Similar expression patterns were seen in large yellow croaker, European sea bass, and Nile tilapia (*Oreochromis niloticus*), with the gills and spleen displaying the highest levels of *mhcII* expression [11,12,31]. The gills and spleen of Atlantic salmon and rainbow trout are the primary immune-related tissues and play a crucial role in capturing antigens, which might be important for activating adaptive immunity [32,33]. MhcII molecules play an irreplaceable role in antigen presentation, which makes MhcII indispensable to the immune system and are found throughout in immune tissues [34].

In order to rule out the likelihood of the antibody interacting with other antigens, Western blotting was employed to look for cross-reactivity between anti-MhcIIα and anti-MhcIIβ Abs (Figure 4). The results demonstrated that the two Abs did not interact with each other. Additionally, it has been proven that the anti-MhcIIα Abs exclusively identify eukaryotically transfected MhcIIα proteins, and the anti-MhcIIβ Abs exclusively identify eukaryotically transfected MhcIIβ proteins (Figure 5 and Figure S1). This guaranteed the anti-MhcII Abs’ accuracy in identifying MhcII^+^ cells.

In mammals, MhcIIα and MhcIIβ molecules are predominantly distributed on DCs, B lymphocytes, macrophages, microglia, and epithelial cells; most of them are APCs [35,36]. Expression of *mhcII* genes and proteins is predominantly found in lymphoid tissues, and functional studies have shown upregulation of *mhcII* gene expression in response to stimulation in vivo or in vitro. However, this is limited with markers for cell types, and further identification of MhcII^+^ cells is necessary [37]. In the study, MhcII^+^ cells were discovered to be more abundantly located in the spleen, gills, PBLs, and kidney, which are immune-associated tissues and similar in yellow catfish (*pelteobagrus fulvidraco*) and Atlantic salmon [37,38]. So far, studies have shown that monocyte macrophages, B-lymphocytes and DCs, and epithelial cells are APCs in teleosts; MhcII is mainly distributed on APCs, whose function is to present processed exogenous antigen to CD4^+^ T cells [22,39,40]. In a previous study, Kurata et al. prepared a specific fMhcIIα Abs, and immunohistochemical staining showed that MhcII^+^ were present in the gills, heart, kidney, spleen, etc. Additionally, these immunopositive cells had rounded or spindly shapes. The morphological characteristics, tissue localization, and the prior literature led to the hypothesis that the MhcII of flounder were expressed by APCs, including DCs, macrophages, B cells, microglia, and epithelial cells [21]. In this study, MhcII was found to co-localize with CD83 and IgM, but not CD4 or CD8, respectively, during FCM (Figure 6) and IIFA (Figure 7) analysis. MhcII was discovered, which was mostly located in DCs and B cells, but not in T lymphocytes. Furthermore, the results showed that MhcII^+^ cells were not totally DCs and B lymphocytes. However, due to the lack of corresponding monocyte and macrophage-specific Abs, we were unable to characterize the other flounder MhcII^+^ cells, which will be the focus of subsequent research.

In mammals, MhcII^+^ cells serve an important function in antigen presentation; MhcII molecules present extracellular antigens such as bacteria, fungi, and parasites. During antigen presentation, MhcII molecules bind to antigenic peptides to form a complex, which is recognized by T-cell receptors and activates CD4^+^ T-cell immune responses [41]. In this study, HIRRV and *E. tarda* of intracellular pathogens and *V. anguillarum* of extracellular pathogens were chosen. Intraperitoneal injections were used to conduct in vivo experiments. Intraperitoneal injections allowed the flounder easy to uptake antigens quickly. Then, the expression of *mhcIIα* and *mhcIIβ* in the SPLs, HKLs, GLs, and PBLs illustrated that strong *mhcIIα* and *mhcIIβ* expressions were detected in HKLs, GLs, and PBLs after three infections. However, early and strong *mhcIIα* and *mhcIIβ* expressions were detected in the SPLs after *E. tarda* infection but not in the infection of *V. anguillarum* (Figure 8A,E). This may be due to the characteristic of pathogen *V. anguillarum* being a kind of opportunistic pathogen, which might provide chronic pathogenicity, while *E. tarda* is a serious pathogenic bacteria that causes intracellular infection and serious pathological change quickly. This is also the case in previous studies; thus, differences in pathogenicity might result in variations in the expression [42]. In miiuy croaker, significant expressions of *mhcIIα* and *mhcIIβ* occurred in the kidney and spleen after 72 h of *V. anguillarum* challenge. The *mhcII* expression in the kidney raised from the beginning of the infection to 12 h, decreased to the lowest point at 36 h, and then increased again to its normal level as control after 72 h [14]. In flounder, the highest *mhcII* expression in SPLs, HKLs, GLs, and PBLs was observed at 24–48 h; most of them regressed to the control at 96 h, except *mhcIIα* expression in HKLs after HIRRV infection (Figure 8C) and *mhcIIα* expression in PBLs after *E. tarda* infection (Figure 8D), which indicated that the infection by *E. tarda* or HIRRV kept for a longer period until the end of the experiment; additionally, infection progress made *mhcIIα* expression upregulated. Similar expression patterns of *mhcIIα* and *mhcIIβ* that were induced by *V. anguillarum* were observed in SPLs, GLs, and HKLs, whereas the two *mhcII* genes showed different patterns in PBLs (Figure 8D,H); this was probably due to the differences in signaling pathways induced by the pathogenic infections. Similarly, in miiuy croaker, after infection of *V. anguillarum, mhcIIα* and *mhcIIβ* genes’ expression patterns were different in the liver and intestine [14]. The *mhcII* expression in GLs and PBLs showed higher growth trends than the study (Figure 8B,D,F,H); the high levels of *mhcII* expression in GLs and PBLs suggested that MhcII^+^ cells responded rapidly to the invasion of pathogenic organisms and indicated that immune cells were involved in trafficking and immune response after antigen stimulation.

From an evolutionary point of view, teleosts, as lower vertebrates, possessed an adaptive immune response: the capacity for an immune response that is antigen-specific. After a teleost is stimulated by antigen, the APCs process the peptide to T cells, which are activated to secrete a variety of cytokines and activate B lymphocytes to produce antigen-specific Abs [35,36,37]. In previous experiments, we demonstrated that MhcII molecules can serve as surface marker molecules for APCs and that the expression of MhcII molecules was significantly upregulated after pathogen stimulation. We also found that turbot MhcII molecules were expressed in B lymphocytes, which could co-localize with CD83, a surface marker molecule of DCs, but not in T lymphocytes, suggesting that MhcII were expressed specifically in APCs. Since the results of the experiments in vivo indicated that GLs and PBLs had the most pronounced upregulation of *mhcII* genes after infection, the circulating tissues were sensitive to capture antigens and might be the place where APC is abundantly accumulated. We further explored in vitro the expression properties of MhcII molecules at the cellular level after stimulation with the pattern antigens LPS, polyI:C, PHA, and ConA, and analyzed their coordinated responses with CD4^+^ T lymphocytes, thus attempting to explain the immune process of teleost MhcII-presenting antigen-activating CD4^+^ T lymphocytes. According to the findings, MhcII^+^/CD83^+^ and MhcII^+^/IgM^+^ cell ratios in the PBLs of flounder were significantly elevated under the stimulation of the thymus-independent antigen LPS (Figure 9B,C), suggesting that B lymphocytes and DCs responded significantly to the stimulation of the thymus-independent antigen. We found that the number of MhcII^+^/CD83^+^ cells in the PBLs of flounder increased significantly with time after LPS stimulation, confirming the response process after antigenic stimulation of flounder on DCs. In zebrafish, the LPS activation of isolated DCs resulted in the upregulated expression of *mhcII* and CD83 [43]. The proportions of MhcII^+^, MhcII^+^/IgM^+^, MhcII^+^/CD83^+^, and CD4^+^ cells in the PBLs of flounder after polyI:C stimulation were significantly elevated at 12–24 h. Similarly, in rainbow trout, polyI:C stimulation significantly upregulated MhcII cell surface expression on IgM^+^ cells in PBLs; in addition, in the polyI:C activation of isolated DCs, the MhcII expression was also significantly upregulated [44,45]. This demonstrates that stimulation via viral organisms is capable of significantly eliciting an immune response in APCs and T lymphocytes [25]. Similarly, the proportions of MhcII^+^, MhcII^+^/IgM^+^, MhcII^+^/CD83^+^, and CD4^+^ cells in PBLs and GLs were significantly increased at 48–72 h after stimulation with the thymus-dependent antigens PHA and ConA. These studies showed different patterns of antigens, all of which elicited the response of MhcII^+^ cells in flounder. The response of MhcII^+^ (Figure 9A,E) and CD4^+^ cells (Figure 9D,H) correlated positively in teleost. MhcII is predominantly distributed in APCs, implying the possibility for presenting antigens to CD4^+^ T cells. Consequently, this result indirectly suggests the existence of an immune process involving the activation of CD4^+^ T cells via antigen delivery via MhcII molecules on APCs present in flounder [46]. However, the process by which fish MhcII presents antigens, the binding of MhcII molecules to CD4 molecules, and the activation process of teleost T lymphocytes remain unclear.

In brief, the present research evaluated the molecular features and tissue location traits of MhcIIα and MhcIIβ, made the polyclonal Abs, revealed that flounder DCs and B lymphocytes expressed MhcII molecules, and investigated the response characteristics of MhcII after pathogenic infection and antigenic stimulation. MhcII responded significantly to exogenous antigens and T cell-dependent antigens, especially playing an essential role in the antigen presentation process, and revealed the existence of the immune process of activation of CD4^+^ T cells via MhcII molecules on APCs. These results will provide solid references to antigen presentation studies in teleost.

## 5. Conclusions

The MhcIIα and MhcIIβ of flounder were transmembrane molecules. They are significantly expressed in immunological tissues and mainly expressed in B lymphocytes and dendritic cells. They did not express in T lymphocytes. The MhcIIα and MhcIIβ responded significantly to exogenous antigens and T cell-dependent antigens. These results will provide an important basis for the study of antigen presentation in teleost.

## Figures and Tables

**Table 1:** Primers.

Primer Name	Primer Sequence(5′−3′)	Application
*mhcIIα*-F	ATGAACGTGAACGTCCTTG	Full-length cloning
*mhcIIα*-R	TCAGCTGCACTCGTTCC	
*mhcIIβ*-F	ATGGCTTCATTCATCCTCAGCTT	
*mhcIIβ*-R	TTAGTGGGAACCAGGATCCCG	
*mhcIIα*CO-F	GGCCATGGCTGATATCGGATCCGAAGGTCTACATGTGGACCGTC(BamHⅠ)	Extracellular domain cloning
*mhcIIα*CO-R	GACGGAGCTCGAATTCGGATCC CGGTCCAACACTGGGCTG(BamHⅠ)	
*mhcIIβ*CO-F	GGCCATGGCTGATATCGGATCC GATGGATTTCTGCATTATACGG(BamHⅠ)	
*mhcIIβ*CO-R	GACGGAGCTCGAATTCGGATCCCTTGTTTCTTTCTGACTCAGGC(BamHⅠ)	
q *mhcIIα*-F	GACGGTGAAGAGATGTGGTT	qPCR
q *mhcIIα*-R	ATCGGACTGGAGGGAGGC	
q *mhcIIβ*-F	AAGTCTGGAGAGAAGATTTCCTGTG	
q *mhcIIβ*-R	GATGAATCCAGCCAGAGATAAGGT	
q*β-actin*-F	GAGGGAAATCGTGCGTGACAT	
q*β-actin*-R	ATTGCCGATGGTGATGACCTG	
*mhcIIα*-GFP-F	ACTCAGATCTCGAGCTCAAGCTTATGAACGTGAACGTCCTTG(Hind Ⅲ)	Eukaryotic transfection
*mhcIIα*-GFP-R	GTCGACTGCAGAATTCGAAGCTTGCTGCACTCGTTCCCTT(Hind Ⅲ)	
*mhcIIβ*-GFP-F	ACTCAGATCTCGAGCTCAAGCTTATGGCTTCATTCATCCTCAGC(Hind Ⅲ)	
*mhcIIβ*-GFP-R	GTCGACTGCAGAATTCGAAGCTTGTGGGAACCAGGATCCCGT(Hind Ⅲ)	

Underlines indicate homologousarmprimers.

## Data Availability

The data in this research are accessible upon request from the corresponding author.

## Supplementary Materials

The following supporting information can be downloaded at: https://www.mdpi.com/article/10.3390/biology12121464/s1, Figure S1: Indirect immunofluorescence results of rabbit anti-flounder MhcIIα and MhcIIβ Abs and HEK293 cell lines transfected with eukaryotic plasmid; Figure S2: Flow cytometry results of the proportion of MhcIIα^+^, MhcIIα^+^/CD4-1^+^, MhcIIα^+^/CD4-2^+^, MhcIIα^+^/IgM^+^, MhcIIα^+^/CD8^+^ cells in peripheral blood, gills, head kidney, and spleen; Figure S3: Flow cytometry results of the proportion of MhcIIβ^+^, MhcIIβ^+^/CD4-1^+^, MhcIIβ^+^/CD4-2^+^, MhcIIβ^+^/IgM^+^ and MhcIIβ^+^/CD8^+^ cells in peripheral blood, gills, head kidney, and spleen.
