# Supplementary material for: Expression and Immune Characterization of Major Histocompatibility Complex in Paralichthys olivaceus after Antigen Stimulation
Source: Biology (Basel). 2023 Nov 24;12(12):1464. doi: 10.3390/biology12121464 (PMC10741117; doi:10.3390/biology12121464)
Supplement: Supplementary file 1 [file biology-12-01464-s001.zip › biology-2691936-supplementary.pdf]

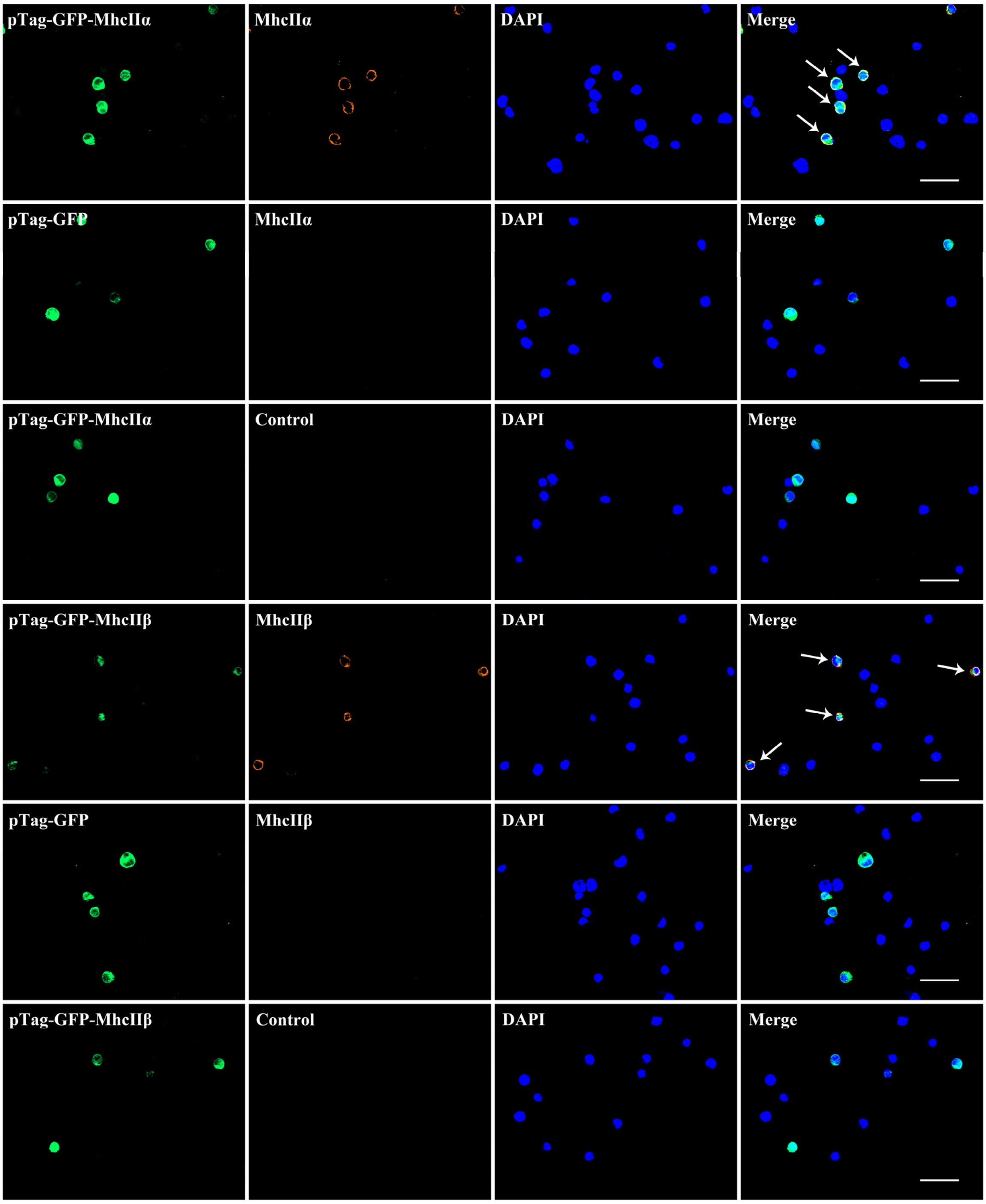

Figure S1. Indirect immunofluorescence results of rabbit anti-flounder MhcIIα and MhcIIβ Abs and HEK293 cell lines transfected with eukaryotic plasmid. Arrows indicate double positive cells. Bar=50 μm.

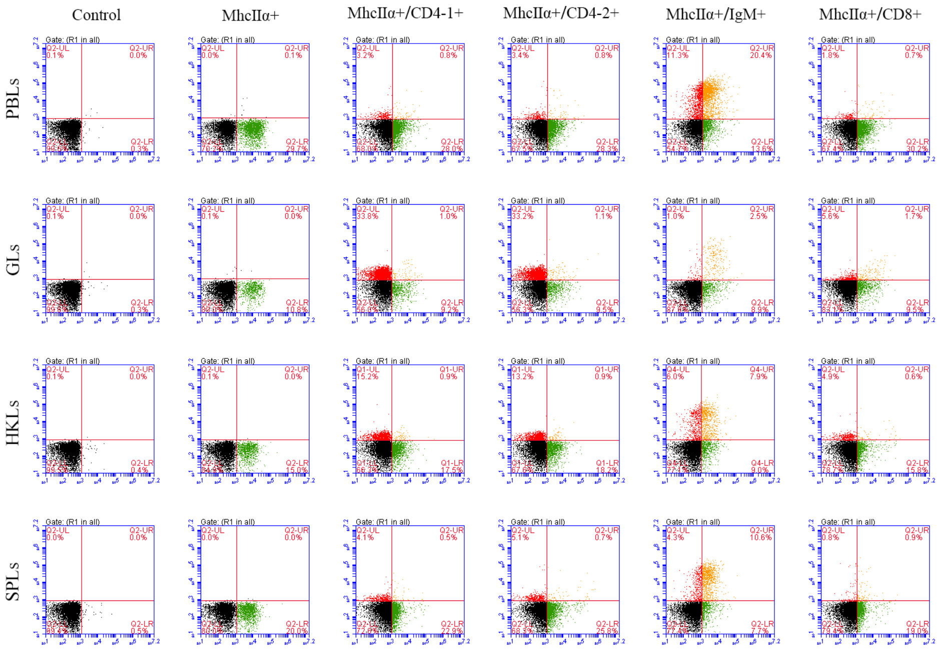

Figure S2. Flow cytometry results of the proportion of MhcIIα^{+}, MhcIIα^{+}/ CD4-1^{+}, MhcIIα^{+}/ CD4-2^{+}, MhcIIα^{+}/ IgM^{+}, MhcIIα^{+}/ CD8^{+} cells in peripheral blood, gills, head kidney, and spleen. In control group, unimmunized rabbit and mouse serum were used as primary antibody.

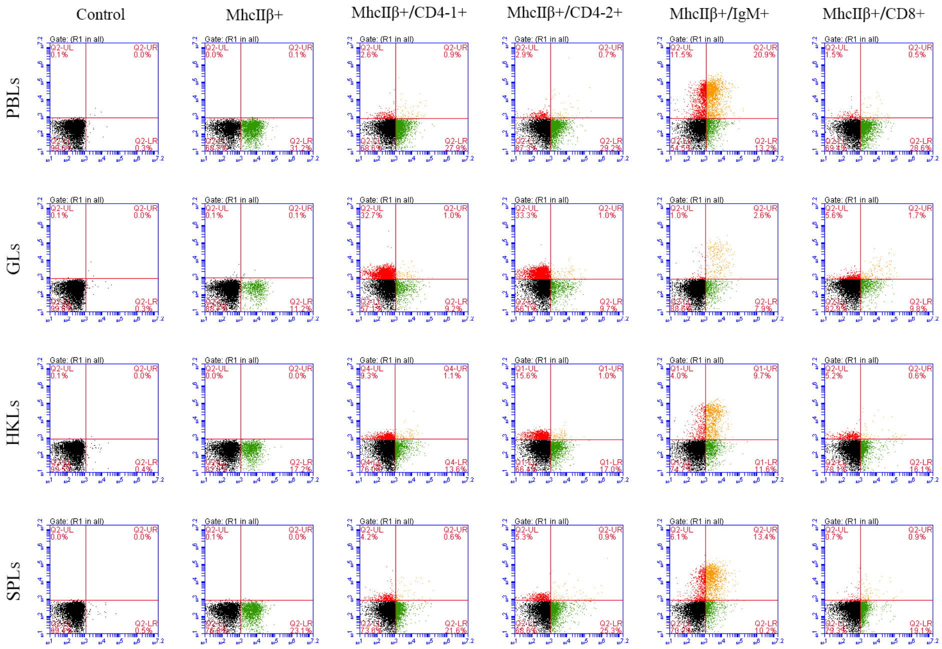

Figure S3. Flow cytometry results of the proportion of MhcIIβ^{+}, MhcIIβ^{+}/ CD4-1^{+}, MhcIIβ^{+}/ CD4-2^{+}, MhcIIβ^{+}/ IgM^{+} and MhcIIβ^{+}/ CD8^{+} cells in peripheral blood, gills, head kidney, and spleen. In control group, unimmunized rabbit and mouse serum were used as primary antibody.
